# Secondary Hirata Syndrome in a Neonate: Practical Approach and Management

**DOI:** 10.1210/jcemcr/luae109

**Published:** 2024-07-01

**Authors:** Alessandro Perri, Simona Fattore, Annamaria Sbordone, Luca Viti, Dario Pitocco, Giovanni Vento

**Affiliations:** Department of Woman and Child Health Sciences, Child Health Area, University Hospital Agostino Gemelli, Fondazione Policlinico Universitario A. Gemelli IRCCS, 00168 Rome, Lazio, Italy; Department of Woman and Child Health Sciences, Child Health Area, University Hospital Agostino Gemelli, Fondazione Policlinico Universitario A. Gemelli IRCCS, 00168 Rome, Lazio, Italy; Department of Woman and Child Health Sciences, Child Health Area, University Hospital Agostino Gemelli, Fondazione Policlinico Universitario A. Gemelli IRCCS, 00168 Rome, Lazio, Italy; Diabetes Care Unit, Department of Translational Medicine and Surgery, Università Cattolica del Sacro Cuore, Fondazione Policlinico Universitario Agostino Gemelli IRCCS, 00168 Rome, Italy; Diabetes Care Unit, Department of Translational Medicine and Surgery, Università Cattolica del Sacro Cuore, Fondazione Policlinico Universitario Agostino Gemelli IRCCS, 00168 Rome, Italy; Department of Woman and Child Health Sciences, Child Health Area, University Hospital Agostino Gemelli, Fondazione Policlinico Universitario A. Gemelli IRCCS, 00168 Rome, Lazio, Italy; Department of Woman and Child Health Sciences, Child Health Area, Catholic University of Sacred Heart Seat of Rome, 00168 Rome, Lazio, Italy

**Keywords:** Hirata, neonatal hypoglycemia, insulin autoimmune syndrome, insulin antibodies, neonate

## Abstract

Hirata disease, also known as insulin autoimmune syndrome (IAS), is a rare cause of hypoglycemia, due to the presence of insulin autoantibodies (IAA) in the circulating blood. These antibodies are immunoglobulin G (IgG), making placental transfer to the fetus possible. To our knowledge, no reports of IAS have been previously described in the neonatal population. We present a case report of hypoglycemia due to a secondary IAS in a neonate and discuss the management and treatment of the disease.

## Introduction

Hypoglycemia is a frequent clinical problem in neonatology (1); it is very common in preterm babies (gestational age [GA] < 37 weeks of pregnancy), but it can also present in a population of “at-risk” term infants. Guidelines for the treatment of hypoglycemia show both how to recognize neonates and children at risk for hypoglycemia and how to treat affected patients (2, 3). Nevertheless, in neonatology, there is a debate over the definition of the thresholds of hypoglycemia and consequently of the treatment of affected babies (4). Neonatal hypoglycemia can present asymptomatically, making maternal history crucial in the management of the neonate. Screening for hypoglycemia in neonates at risk is mandatory, as a prolonged state of hypoglycemia and wide fluctuation of blood glucose levels are associated with poor neurodevelopmental outcomes (5).

Hirata disease, also known as insulin autoimmune syndrome (IAS), is a rare cause of hypoglycemia, due to the presence of insulin autoantibodies (IAA) in the circulating blood. IAS is usually a disease of adult life, and it is more common in Japan, being the third most common cause of hypoglycemia after insulinoma and extrapancreatic tumors (6). While this disease is rare in the White population, it can present in women at risk, being associated with the presence of the human leukocyte antigen HLA-DR4. The genetic predisposition and the usage of specific drugs during pregnancy can lead to the disease and the production of IAA. These antibodies are IgG, allowing for placental transfer to the fetus. To our knowledge, no reports of IAS have been previously described in the neonatal population. We present a case report of hypoglycemia due to IAS in a neonate. We discuss the management and treatment of the disease.

## Case Presentation

We present the case of a neonate born at term at GA 38 + 4 weeks by vaginal delivery. Birth weight was 2960 g (29°pc, −0.53 *z* score (7)), head circumference was 34 cm (59°pc, 0.24 *z* score), and length was 49 cm (50°pc, 0.00 *z* score). Apgar index was 9 at 1 minute and 10 at 5 minutes. Prenatal ultrasounds were normal. No signs of fetal hypoglycemia were detected during pregnancy. The pregnancy was complicated by Hashimoto thyroiditis treated with levothyroxine; celiac disease; VII factor deficiency; and COVID infection during the second trimester of pregnancy. The mother did not have gestational diabetes. At the 18th week of pregnancy, the mother was hospitalized because of severe hypoglycemia episodes. These episodes started a month and a half before the hospitalization. Hirata disease was diagnosed and treated with a glucose infusion, a specific diet, and prednisone. The trigger for the disease was identified as α-lipoic acid supplementation (600 mg twice a day). The medication was discontinued after the diagnosis of Hirata disease.

## Diagnostic Assessment

After birth, the neonatal blood glucose levels were monitored. During the hospital stay, the baby had several hypoglycemic episodes. Hypoglycemic episodes were defined as a glycemic value of less than 47 mg/dL (2.6 mmol/L) during the first 48 hours of life and less than 60 mg/dL (3.3 mmol/L) after 48 hours of life for more than 10 minutes. The most severe episode occurred on the third day of life, when the minimum glycemic value was 35 mg/dL.

Screening for hypoglycemia was started at birth, and the neonate was observed in the special care baby unit. When the baby developed hypoglycemia, he was treated with a “feeding strategy.” The baby was fed 10 meals per day to reduce fasting time. The glucose intake needed not to develop a severe hypoglycemia episode was 14 g/kg/day (778 mmol/kg/day). A continuous glucose monitoring system (CGMS) (Medtronic paradigm VEO, ENLITE sensor) was used to monitor the glucose levels. Hypoglycemia episodes lasted no more than 30 minutes. We classified the episodes as mild: blood glucose less than 60 mg/dL (3.3 mmol/L, normal value of blood glucose: 60-180 mg/dL; 3.3-9.9 mmol/L), and severe: blood glucose less than 47 mg/dL (2.6 mmol/L) (8). Even when the hypoglycemia was severe, rarely was the blood glucose level lower than 40 mg/dL (2.2 mmol/L) (we registered 3 episodes during the whole hospitalization). All the hypoglycemic episodes were asymptomatic. Furthermore, they were usually unpredictable, even if we noticed that sometimes these episodes followed mild early postprandial hyperglycemia. IAA were measured at 61 IU/mL (normal range <10 IU/mL). The baby had a normal blood pH and a normal lactate level. The baby was discharged home when all the following criteria were met: no severe hypoglycemia and no more than 2 mild hypoglycemia episodes were detected during a period of 72 hours, and the parents were able to manage CGMS.

## Treatment

The baby did not need any specific treatment except for the adjustment of glucose intake based on glycemic values. He received a specific “feeding strategy” consisting of 10 meals per day.

## Outcome and Follow-up

Discharge was possible on the baby’s 26th day of life. A fasting trial of 6 hours was performed, and it did not show a blood glucose concentration under 60 mg/dL (3.3 mmol/L). A strict follow-up plan to be followed after discharge was shared with the parents. The collected data showed that during the hospitalization, the daily hypoglycemic episodes were 1.15 (±0.06), while during the follow-up, the daily hypoglycemia episodes were 0.29 (±0.07); data are presented as median (SD). A follow-up program was arranged consisting of a review of the glycemic trend and clinical assessment every 2 weeks. Glycemic trend normalized from the 48th day of life. Dosage of IAA was repeated on the 55th day of life, and results were negative. The CGMS was removed. The baby will undergo a neurological follow-up for the first year of life.

## Discussion

The pathogenesis of Hirata disease involves the interaction between genetic and environmental factors (ie, infections, drugs) (9, 10). The onset of IAS in the neonatal period can be secondary to the presence of IAA, due to the maternal-fetus transplacental transfer of antibodies. The mechanism is the following: Insulin is normally secreted by pancreatic β cells in response to rising plasma glucose concentration. IAA bind to the circulating insulin, leading to a reduction in its physiological effect. Transient hyperglycemia occurs, and more insulin is secreted. In a second phase, the dissociation of insulin from immune complexes with autoantibodies creates a relative excess of insulin, resulting in the lowering of the glycemia under the threshold of hypoglycemia.

In this case, the trigger for the disease was identified as α-lipoic acid supplementation (600 mg twice a day). This medication contains a sulfhydryl group, which is known to be responsible for the immune reaction leading to the production of IAA.

The finding of IAA in a blood sample leads to the diagnosis of IAS. IAA can be detected with laboratory tests: precipitation with polyethylene glycol, followed by an insulin assay (11). The insulin-to-C-peptide ratio is an ancillary method. The normal insulin to C-peptide ratio is less than 1. In the physiological setting, insulin and C-peptide are secreted by the pancreatic β cells simultaneously, then insulin is rapidly (half-life of 5-10 minutes) metabolized by the liver, while C-peptide is slowly (half-life of 30-35 minutes) eliminated by the kidneys. In IAS, IAA binding delays the half-life of insulin.

The removal of C-peptide remains unchanged, making the ratio greater than 1 (12-14). Furthermore, in IAS, C-peptide levels may be raised as a consequence of the interference with the available immunoassays; proinsulin cross-reactivity in some C-peptide immunoassays can lead to falsely increased C-peptide results (15). IgG antibodies are eliminated from the circulating blood of the baby in 3 to 6 months, so the hypoglycemia episodes could appear in that time window.

No guidelines can be found in literature for the management of a neonate with secondary IAS. In the adult population, patients are advised to eat frequently to prevent fasting and, in severe cases, continuous enteral or parenteral feeding may be needed (16, 17). Treatment is also an option; therapies with corticosteroids, calcineurin inhibitors (rituximab, cyclophosphamide, and mycophenolate mofetil) are described. Diazoxide and octreotide have also been used as they can decrease insulin production from β cells (9).

**Figure 1.**
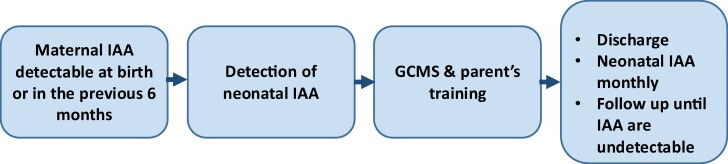
A proposal for clinical management of a neonate born from a mother with insulin autoantibodies (IAA) during pregnancy. For all these neonates, a screening for the detection of hypoglycemia is suggested. We propose a screening of at least 72 hours. The evaluation of the glycemia after the rising of the glycemia following a meal (breastfeeding should be effective after 24-48 hours from birth) is mandatory. Glycemia can be detected every 3 hours in the asymptomatic patient.

We propose a practical approach for the neonate at risk for IAS and severe hypoglycemia, summarized in Fig. 1. When there is a maternal history of IAS, the neonatologist should verify when the IAA were detected during the pregnancy. The neonate should be screened for hypoglycemia and, if the maternal IAA were detectable during the last 4 months of pregnancy, IAA should be tested in a neonatal blood sample.

Hypoglycemia episodes can be treated following standard protocols. The detection of neonatal IAA needs specific management. Hypoglycemia can be detected for a long period after birth. To reduce the number of heel-prick blood samples, we suggest using a CGMS. The number of calibration procedures depends on the brand of the device, but varies from 1 every 12 hours to 0.

**Figure 2.**
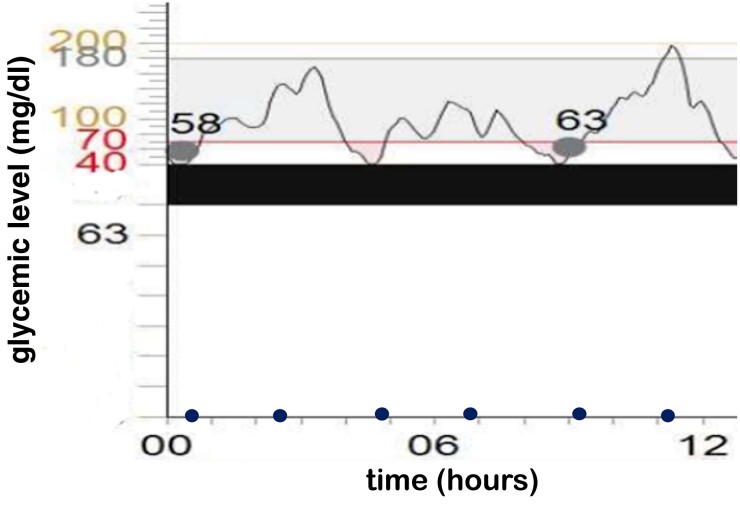
Glycemic levels. The x-axis represents the hour; the y-axis represents blood glucose levels. Time of the meals are displayed with blue dots. Dots crossing the graphic are the calibration of the continuous glucose monitoring system.

This is the first time an IAS has been described in the neonatal period. Hypoglycemia episodes in this patient have been both mild and severe, and the neonate was asymptomatic. Glycemic levels are shown in Fig. 2. The neonatal staff trained the parents to manage the CGMS. Discharge is possible when the neonate does not need parenteral glucose intake, there is no severe hypoglycemia, and no more than 2 mild hypoglycemia episodes can be detected during a 72-hour period; a fasting test results in no hypoglycemia episodes.

We propose a follow-up every 14 days, in which the glycemic trend, hypoglycemia episodes, and the feeding routine of the baby are evaluated. The IAA should be measured every month until they are undetectable. Once this is verified, the follow-up and the CGMS can be ended.

The management of the hypoglycemia in this particular patient was challenging. Our medical team decided not to start the infusion of glucose as severe hypoglycemia episodes were sporadic, asymptomatic, responsive to feeding, and they occurred during the first 72 hours of life. Some episodes of mild hypoglycemia were observed before discharge. These episodes could not have been detected with a normal screening (the CGMS detected mild hypoglycemia following the meals, but not in the preprandial phase); they were asymptomatic; they lasted less than 20 minutes, or, when the duration was longer than 20 minutes, they were responsive to supplementary meals.

In conclusion, IAP is a rare cause of hypoglycemia. It is a disease of adults, and it is reversible after the removal of its trigger. IAA can be transferred transplacentally to the fetus during pregnancy, and the management of the neonate can be challenging. We propose a practical regimen for the management of the condition in the neonatal population.

## Learning Points

- Insulin autoantibodies are IgG. They can cross the placenta, causing Hirata syndrome in the neonate.
- In the neonate, CGMS is more effective in detecting hypoglycemic episodes compared to the standard point-of-care procedure.
- It is reasonable for these newborns to be monitored until antibodies become negative.

## Data Availability

Original data generated and analyzed for this case report are included in this published article.

## Abbreviations

CGMS: continuous glucose monitoring system
IAA: insulin autoantibodies
IAS: insulin autoimmune syndrome
IgG: immunoglobulin G
